# Wi-Fi Fingerprint-Based Indoor Localization Method via Standard Particle Swarm Optimization

**DOI:** 10.3390/s22135051

**Published:** 2022-07-05

**Authors:** Jin Zheng, Kailong Li, Xing Zhang

**Affiliations:** 1 School of Architecture and Art, Central South University, Changsha 410083, China; 2 College of Electrical and Information Engineering, Hunan University, Changsha 410082, China; leekl@hnu.edu.cn (K.L.); xing_zx1@hnu.edu.cn (X.Z.)

**Keywords:** Wi-Fi fingerprint, indoor localization, particle swarm optimization, location estimation

## Abstract

With the continuous development and improvement in Internet-of-Things (IoT) technology, indoor localization has received considerable attention. Particularly, owing to its unique advantages, the Wi-Fi fingerprint-based indoor-localization method has been widely investigated. However, achieving high-accuracy localization remains a challenge. This study proposes an application of the standard particle swarm optimization algorithm to Wi-Fi fingerprint-based indoor localization, wherein a new two-panel fingerprint homogeneity model is adopted to characterize fingerprint similarity to achieve better performance. In addition, the performance of the localization method is experimentally verified. The proposed localization method outperforms conventional algorithms, with improvements in the localization accuracy of 15.32%, 15.91%, 32.38%, and 36.64%, compared to those of KNN, SVM, LR, and RF, respectively.

## 1. Introduction

In recent years, Internet-of-Things (IoT) technology, an extension of the Internet that envisions connecting all devices to the Internet for communications, is developing rapidly and is expected to radically transform education, healthcare, smart home, manufacturing, commerce, and transportation, etc. It is essential for transforming the world into a smart world, wherein localization of devices or terminals is an indispensable aspect [1,2,3]. Although the global positioning system (GPS) satisfies the wide requirements of outdoor scenes, it performs poorly and has very limited usage in indoor scenes [4]. There exist numerous requirements and challenges in the development of indoor localization.

According to the signal source, indoor-localization technologies can be divided into external and natural signal sources. External signal sources mainly include Wi-Fi [5], Bluetooth [6], ultra-wide band (UWB) [7], visible light [8], ZigBee [9], computer vision [10], and radio-frequency identification (RFID) [11]. By contrast, indoor localization technology based on natural signal sources primarily relies on the sensors of terminal devices to achieve localization, including inertial measurement units (IMU) [12] and geomagnetics [13], etc. The list of these technologies can be extended as technology develops. For example, Long Range (LoRa), originally developed for long-range communication with a high link budget, can also be employed for indoor localization [14].

Among these, UWB-based indoor-localization technology offers the advantages of high accuracy and simple localization methods; however, it relies on additional deployment devices and incurs a high cost [15]. A localization system based on vision utilizes high-precision computer vision technology, but it can only spread within the line of sight and requires high hardware cost and complex computation [10]. The main principle of RFID localization [16] is to perform non-contact communication transmission using the spatial coupling characteristics of the radio frequency. Passive RFID equipment is cheap but has a small transmission range, whereas active signals have wide coverage but high hardware costs. However, its localization accuracy is inadequate. The IMU localization system uses an accelerometer, gyroscope, magnetometer, and other sensors of the terminal equipment to perform navigation calculations; however, the localization accuracy is limited by hardware devices and inevitably produces cumulative errors [17], which require continuous calibration with external information. For the LoRa, the received signal strength (RSS) distance method, an RSS-based logarithmic path loss model, could be adopted for indoor localization. RSS values are used to calculate the location of an object according to the principle of trilateration [14]. Further, Wi-Fi, ZigBee, and Bluetooth are wireless-sensor-network technologies based on IEEE 802 standards, featuring low power consumption and low cost [18]. The ranging principle is mainly based on geometric constraints and signal-strength feature matching. Zigbee-based localization measures the distance between the unknown and reference points in advance, and the signal has a low transmission rate and a short transmission distance. Moreover, Bluetooth and Wi-Fi are supported in most terminal devices, but the range of Bluetooth signal communication is limited, and the localization accuracy is inadequate, with a large time delay. In contrast, Wi-Fi signal transmission rate is fast, its localization range is wide, and equipment deployment is easy.

In the field of indoor localization, Wi-Fi fingerprint-based localization is a current mainstream method [15]. However, it is limited by the volatility of Wi-Fi signal, which makes offline data not reliable enough, and it is difficult to achieve stable high-accuracy localization. Therefore, this study focused on the accuracy improvement of Wi-Fi fingerprint-based localization, adopting a robust localization model [19] and utilizing the standard particle swarm optimization (SPSO) algorithm [20] to determine the optimal location estimation. The main contributions of this study are as follows:

- A two-panel fingerprint homogeneity model was adopted to characterize fingerprint similarity. In addition to considering both the real distance and direction difference of two fingerprints, this study proposes another combination, Euclidean metric and cosine distance, which was used in the system for a more robust performance.
- An effective application of a standard particle swarm optimization (SPSO) algorithm for Wi-Fi fingerprint-based indoor localization is proposed to improve the localization accuracy.
- Experiments on data sets and tests were conducted in a real-world environment and the results were compared with those obtained using other classical localization methods, thereby verifying the effectiveness of the proposed localization method.

The remainder of this paper is organized as follows. In Section 2, related work is briefly reviewed. Section 3 describes the proposed localization system in detail. In Section 4, the field experiments conducted to examine the proposed algorithm are described, followed by the conclusions in Section 5.

## 2. Related Work

Wi-Fi technology, as an important sensor in IoT, has been utilized in many areas of indoor scenes. For an interesting example, detecting motion in a room or detecting when a potential user approaches a Wi-Fi-enabled device are important applications of Wi-Fi sensing, and of interest in areas such as system wake up and environment monitoring [21]. The suitability of using Wi-Fi to sense fire, another potential application, was demonstrated in [22], which proved that there is a direct relationship between flame and channel state information (CSI) signatures. Indoor localization is also a significant application area of Wi-Fi technology.

### 2.1. Wi-Fi-Based Indoor Localization

Wi-Fi-based indoor-localization technology offers the advantages of wide signal coverage, relatively mature equipment, easy deployment (it needs no additional sensor equipment), low cost, strong applicability, and expansibility [23]. Therefore, indoor-localization technology based on Wi-Fi has high research value and broad application prospects. Wi-Fi-based indoor-localization technology includes the following two types:

- **Triangulation method.** This method relies on the measurement of distance. Thereafter, the location estimation is obtained through geometric calculation. Classical triangulation localization methods [24] include time of arrival (TOA), time difference of arrival (TDOA), and angle of arrival (AOA). TOA is a measurement method to calculate the distance between the terminal device and the Wi-Fi access point (AP) by recording the unidirectional or bidirectional arrival time of Wi-Fi signals between the their terminals. However, it requires the precise synchronization of the time stamps at the transmitter and receiver. TDOA uses the characteristic that two focal distances on a hyperbola remain fixed. Based on the arrival time difference between the terminal device and different APs, using the hyperbolic equation, the location of the localization point can be solved. In contrast to TOA, TDOA reduces the time synchronization requirement, but accurate time measurement is a limiting factor. Moreover, it is susceptible to non-line-of-sight (NLOS) problems. AOA involves obtaining positions through azimuth angle measurements. It eliminates the need for accurate time synchronization between devices and requires a small number of base stations. However, it measures the signal transmission angle, which requires localization equipment carrying an antenna array device, thereby increasing the difficulty of its popularization.
- **Wi-Fi fingerprint-based method.** This method utilizes the mapping correlation between Wi-Fi signal characteristics and physical locations. In the ideal localization environment, each physical location should have a unique and distinguishable fingerprint [23]. Generally, in this method, an indoor-location area is divided into a series of discrete grid spaces in advance to obtain the radio fingerprint map. Further, in the Offline phase, the Wi-Fi received signal strength (RSS) from different access points (APs) are collected on each reference point (RPs) of discrete grid points. Consequently, combining the physical coordinates of RPs, the fingerprint database is constructed. Thus, a received Wi-Fi fingerprint can determine the most similar fingerprint of database in the Online phase, and, subsequently, the corresponding coordinate can be estimated.

By contrast, Wi-Fi fingerprint-based indoor-localization technology is not affected by NLOS and does not require the location information of APs. It has been extensively studied since it was proposed by Bahl [25]. Moreover, many methods have been proposed, which can be divided into deterministic and probabilistic methods.

- **Deterministic methods.** These algorithms directly use the one-to-one mapping relationship between Wi-Fi fingerprint and physical location and estimate the unknown position based on the closest fingerprint location in signal space. K-nearest neighbor (KNN) [25] is one such example. The method is to determine the K most similar Wi-Fi fingerprints from a database using the Euclidean distance, and then calculate the average of the K corresponding physical locations. Ma et al. [26] improved the KNN algorithm and proposed the WKNN algorithm, which used a weighted average for location estimation. Neural network (NN) algorithms [27], such as the multi-layer neural network [28], have also been applied to Wi-Fi fingerprint-based localization but with high computational cost. NN obtains the mapping relationship between a fingerprint and physical position after considerable training, then uses it to predict the unknown position. Certain other, more deterministic, algorithms such as support vector machine [29], random forests [30] and linear discriminant analysis [31] are also used in localization.
- **Probabilistic methods.** Contrary to deterministic algorithms, probabilistic methods employ the probability density function, which characterizes changes in RSS. The key is to predict the possibility of relationships between real-time data and the coordinates of RPs. Horus [32] estimated the unknown position using a probabilistic model considering the signal distribution in the site. Bayesian network [33], expectation maximization [34], Gaussian process [35] and conditional random field [36] are also effective probabilistic algorithms.

In addition, some Wi-Fi fingerprint-based indoor localization frameworks have been proposed. Ref. [37] proposed a Wi-Fi localization framework via fingerprint clustering and adaptive KNN based on fusion fingerprints. They cluster offline fingerprints via the Gaussian mixture model (GMM) to divide the localization area into several subareas. In addition, a random-forest-based subarea classifier is trained by the offline data and corresponding subarea labels used for the online localization. The authors of [19] focused on indoor Wi-Fi fingerprint localization in multistory buildings and proposed a novel floor-identification module with a Wi-Fi-fingerprint-graph representation and a fingerprint graph attention mechanism, to confine the search scope to a specific floor. The two-panel fingerprint-homogeneity graph adopted is a novel mehod to gauge the similarity of Wi-Fi fingerprints robustly.

### 2.2. Particle Swarm Optimization

The particle swarm optimization (PSO) algorithm originated from the study of foraging behavior of birds and other social animals and is a type of biological evolutionary algorithm first proposed by Eberhart and Kennedy [38]. It is a random optimization technique in which the potential solution of the problem is represented by the position of the particles in the swarm. Specifically, a particle is an individual in a bird swarm, and the optimal solution is where the bird looks for the target food. According to the best foraging result of individual birds (historical optimal solution of each particle, pbest) and the best foraging result of the bird swarm (historical optimal solution of the particle swarm, gbest), every bird flies towards the best position and explores the best foraging position. This is calculated using the fitness function value of each particle, and the flight direction is determined to adjust the direction of convergence. Thus, after several iterations, the optimal foraging position (global optimal solution) can be explored by using a bird swarm (particle swarm).

In the standard particle swarm optimization (SPSO) algorithm [20], let the solution space be S with *D* dimensions, and the boundary of each dimension be $\left[x_{d\min},x_{d\max}\right]$. Suppose that the number of particles is $N_{ps}$ and the maximum iteration is *T*. The rules of position updating in the SPSO algorithm are expressed as

(1) $$S=\{x\in R^{D}|x_{d}\in \left[x_{d\min},x_{d\max}\right],d=1,2,\ldots ,D\}$$

(2) $$v_{id}^{t+1}=\omega v_{id}^{t}+c_{1}r_{1}\left(pbest_{id}^{t}-x_{id}^{t}\right)+c_{2}r_{2}\left(gbest_{d}^{t}-x_{id}^{t}\right)$$

(3) $$x_{id}^{t+1}=x_{id}^{t}+v_{id}^{t+1}$$

where $i=1,2,\ldots ,N_{ps}$ represents the index of each particle, and $v_{i}^{t}=\left[v_{i1}^{t},v_{i2}^{t},\ldots ,v_{iD}^{t}\right]$ and $x_{i}^{t}=\left[x_{i1}^{t},x_{i2}^{t},\ldots ,x_{iD}^{t}\right]$ are the velocity and position variables of the *i*th particle in the *t*th iteration, respectively. To control the particles in space S, the velocity $|v_{id}^{t}|$ is limited to the maximum $v_{\max}^{}$. Meanwhile, for the *t*th iteration, each particle has a historical optimal position: ${\mathrm{pbest}}_{i}^{t}=\left[pbest_{i1}^{t},pbest_{i2}^{t},\ldots ,pbest_{iD}^{t}\right]$, and ${\mathrm{gbest}}^{t}=\left[gbest_{1}^{t},gbest_{2}^{t},\ldots gbest_{D}^{t}\right]$ denotes the historical optimal position of the entire particle swarm. Further, $c_{1}$ and $c_{2}$ denote the acceleration factor and $r_{1}$ and $r_{2}$ are random numbers in the range [0, 1]. In addition, ω is the inertia weight, and its initial and final values are $\omega_{init}$ and $\omega_{end}$ for $T_{\max}$ iterations, respectively, which can be defined as follows:

(4) $$\omega^{t}=\omega_{init}+\left(\omega_{end}-\omega_{init}\right)\left(\frac{T_{\max}-t}{T_{\max}}\right)$$

PSO has been applied in various fields of practical engineering owing to its advantages of fast convergence and nonlinearity. The essence of indoor localization involves determining the optimal location estimation; therefore, the SPSO algorithm is feasible for Wi-Fi indoor localization. However, few scholars have discussed the application of the PSO algorithm in Wi-Fi localization technology, such as combining the AP selection strategy [39], the wireless-signal-propagation model [40], and triangulation localization [41]. In addition, Bi et al. [42] represented the fingerprint of each particle using the inverse distance weighted algorithm to find the optimal location estimation using PSO. Li et al. [43] combined PSO and an artificial neural network (ANN) to reduce the localization error and shorten the prediction time. However, at present, the PSO algorithm has yet to be sufficiently developed and explored in the design of indoor-localization models based on Wi-Fi-fingerprint.

## 3. System Overview

**Figure 1:**
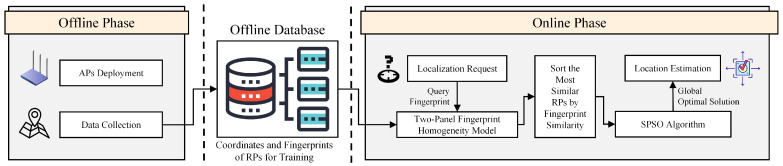
Schematic of the localization system.

A schematic of the proposed localization system is shown in Figure 1. A two-panel fingerprint homogeneity model was used to characterize fingerprint similarity. Subsequently, a fitness function was provided for SPSO, and the optimal solution can be obtained. This is the optimal location estimation for query data. The process is described in detail in the following sections.

### 3.1. Preliminary

For a clear description, certain primary notations are defined here and listed in Table 1.

Given sets of RPs ${\mathrm{RP}}_{train}=\left\{{RP}_{train\;1},{RP}_{train\;2},\ldots {RP}_{train\;N_{tr}}\right\}$ and APs $\mathrm{AP}=\{{AP}_{1},{AP}_{2},\ldots {AP}_{M}\}$, suppose that *M* APs are deployed in the indoor environment, and $N_{tr}$ RPs are selected as signal collection points in the offline phase. Consequently, each RP has a coordinate ${RP}_{train\;i}=\left(x_{train\;i},y_{train\;i}\right)$ and a Wi-Fi fingerprint ${Fin}_{train\;i}$ (as in Equation (5)). The entire Wi-Fi fingerprint in the offline phase is denoted by Equation (6).

(5) $${Fin}_{train\;i}=\left(RSS_{train\;i}^{AP\;1},RSS_{train\;i}^{AP\;2},\ldots ,RSS_{train\;i}^{AP\;M}\right)$$

(6) $$\begin{array}{cc} {\mathrm{Fin}}_{train} & ={\left(Fin_{train\;1},Fin_{train\;2},\cdots ,Fin_{train\;N_{tr}}\right)}^{T}\\ \; & =\left[\begin{array}{cccc} RSS_{train\;1}^{AP\;1} & RSS_{train\;1}^{AP\;2} & \cdots & RSS_{train\;1}^{AP\;M}\\ RSS_{train\;2}^{AP\;1} & RSS_{train\;2}^{AP\;2} & \cdots & RSS_{train\;2}^{AP\;M}\\ \cdots & \cdots & \ddots & \vdots \\ RSS_{train\;N_{tr}}^{AP\;1} & RSS_{train\;N_{tr}}^{AP\;2} & \cdots & RSS_{train\;N_{tr}}^{AP\;M} \end{array}\right] \end{array}$$

where $RSS_{train\;i}^{AP\;j}$ is the RSS of *i*th RP from *j*th AP ($i=1,2,\ldots ,N_{tr};j=1,2,\ldots ,M$). Similarly, in the online phase, a series of (such as $N_{te}$) query fingerprints ${\mathrm{Fin}}_{query}$ are collected when some users make a location request. In this study, each of these was denoted as a test fingerprint ${Fin}_{test\;i}$:

(7) $${Fin}_{test\;i}=\left(RSS_{test\;i}^{AP\;1},RSS_{test\;i}^{AP\;2},\ldots ,RSS_{test\;i}^{AP\;M}\right)$$

(8) $$\begin{array}{cc} {\mathrm{Fin}}_{test} & ={\left(Fin_{test1},Fin_{test2},\cdots ,Fin_{testN_{te}}\right)}^{T}\\ \; & =\left[\begin{array}{cccc} RSS_{test\;1}^{AP\;1} & RSS_{test\;1}^{AP\;2} & \cdots & RSS_{test\;1}^{AP\;M}\\ RSS_{test\;2}^{AP\;1} & RSS_{test\;2}^{AP\;2} & \cdots & RSS_{test2}^{AP\;M}\\ \cdots & \cdots & \ddots & \vdots \\ RSS_{test\;N_{te}}^{AP\;1} & RSS_{test\;N_{te}}^{AP\;2} & \cdots & RSS_{test\;N_{te}}^{AP\;M} \end{array}\right] \end{array}$$

where $N_{te}$ denotes the number of test fingerprints. Correspondingly, the actual coordinates are ${RP}_{test\;i}=\left(x_{test\;i},y_{test\;i}\right),{RP}_{test\;i}\in {\mathrm{RP}}_{test}=\left\{{RP}_{test\;1},{RP}_{test\;2},\ldots {RP}_{test\;N_{te}}\right\},i=1,2,\ldots ,N_{te}$.

### 3.2. Two-Panel Fingerprint-Homogeneity Model

In Wi-Fi fingerprint-based indoor localization system, similarity characterization is essential for the test fingerprint ${Fin}_{test\;i}$ to match the *K* most similar training fingerprints $Fin_{{}_{train}}^{sim\;k},\left(k=1,2,\ldots ,K\right)$ in the offline database ${\mathrm{Fin}}_{train}$. Generally, it is expressed in terms of Euclidean distance; the closer the distance, the more similar it is to the fingerprints. Then, the location estimation can be calculated using Equation (9).

(9) $$\left(x,y\right)=\left(\frac{\sum_{k=1}^{K}x_{train\;k}}{K},\frac{\sum_{k=1}^{K}y_{train\;k}}{K}\right)$$

To further constrain the bias in fingerprint similarity characterization, a two-panel fingerprint-homogeneity model [19] was adopted to gauge the similarity of different fingerprints. In contrast to [19], for the first panel, Euclidean distance was used to gauge the homogeneity of different data. Further, the cosine distance was used to reflect the divergence of different vectors from a directional aspect in another panel. For vectors with the same dimension *n*, the two distances are denoted by Equations (10) and (11), and the similarity can be expressed as Equations (12) and (13).

(10) $${dis}_{\mathrm{Euc}}\left(v_{1},v_{2}\right)={∥v_{1}-v_{2}∥}_{2}=\sqrt{\sum_{i=1}^{n}{\left(v_{1i}-v_{2i}\right)}^{2}}$$

(11) $$dis_{\cos}\left(v_{1},v_{2}\right)=1-\frac{v_{1}\cdot v_{2}}{\|v_{1}\left\|\cdot \right\|v_{2}\|}$$

(12) $$sim_{\mathrm{Euc}}\left(v_{1},v_{2}\right)=10^{-dis_{\mathrm{Euc}}\left(v_{1},v_{2}\right)}$$

(13) $$sim_{\cos}\left(v_{1},v_{2}\right)=10^{-dis_{cos}\left(v_{1},v_{2}\right)}$$

For a specific test fingerprint ${Fin}_{test}$ and a specific training fingerprint ${Fin}_{train}$, their Euclidean and cosine distances are denoted by Equations (14) and (15). Next, the corresponding location coefficients (as weights) were obtained based on the two distances, as Equations (16) and (17).

(14) $$dis_{\mathrm{Euc}}\left({Fin}_{test},{Fin}_{train}\right)=\sqrt{\sum_{j=1}^{M}{\left(RSS_{test}^{AP\;j}-RSS_{train}^{AP\;j}\right)}^{2}}$$

(15) $$dis_{\cos}\left({Fin}_{test},{Fin}_{train}\right)=1-\frac{\sum_{j=1}^{M}RSS_{test}^{AP\;j}\cdot RSS_{train}^{AP\;j}}{\sqrt{\sum_{j=1}^{M}{\left(RSS_{test}^{AP\;j}\right)}^{2}}\sqrt{\sum_{j=1}^{M}{\left(RSS_{train}^{AP\;j}\right)}^{2}}}$$

(16) ωEuck=10^[−disEuc(Fintest,FintrainsimEuck)]

(17) ωcosk=10^[−discos(Fintest,Fintrainsimcosk)]

Actually, if only one panel of the two-panel fingerprint-homogeneity model or other distance metrics were used, the localization results would be affected. Different combinations will result in different performances. The details are discussed in Section 4.

### 3.3. SPSO Algorithm for Localization

To obtain the optimal predicted location of $Fin_{test\;i}$, the optimal value of the parameter *K* and the coordinate (x,y) must be solved. The SPSO algorithm can be used for this purpose. The target fitness function is defined by Equation (18).

(18) $$\begin{array}{cc} f(x,y,K)= & \sqrt{{\left(x-\sum_{k=1}^{K}\frac{\omega_{\mathrm{Euc}\;k}}{\sum_{k=1}^{K}\omega_{\mathrm{Euc}\;k}}x_{train}^{sim_{\mathrm{Euc}}k}\right)}^{2}+{\left(y-\sum_{k=1}^{K}\frac{\omega_{\mathrm{Euc}\;k}}{\sum_{k=1}^{K}\omega_{\mathrm{Euc}\;k}}y_{train}^{sim_{\mathrm{Euc}}k}\right)}^{2}}\\ \; & +\sqrt{{\left(x-\sum_{k=1}^{K}\frac{\omega_{\cos\;k}}{\sum_{k=1}^{K}\omega_{\cos\;k}}x_{train}^{sim_{\cos}k}\right)}^{2}+{\left(y-\sum_{k=1}^{K}\frac{\omega_{\cos\;k}}{\sum_{k=1}^{K}\omega_{\cos\;k}}y_{train}^{sim_{\cos}k}\right)}^{2}} \end{array}$$

At each iteration, the minimum fitness value of the particle and particle swarm were determined. Finally, the optimal location estimation was obtained. The specific procedure is summarized in Algorithm 1. In addition, the mathematical model of the algorithm can be found at the link: https://github.com/Kiron666/SPSO_2P (accessed on 30 June 2022).
**Algorithm 1** The algorithm procedure of localization.**Input:**    The offline fingerprints data ${\mathrm{Fin}}_{train}$ and the coordinates data ${\mathrm{RP}}_{train}$;    The query fingerprint ${\mathrm{Fin}}_{query}$;**Output:**    The location estimation of the query fingerprint.1:Offline data collection, and obtain the training data ${\mathrm{Fin}}_{train}$, ${\mathrm{RP}}_{train}$;2:Obtain the query data ${\mathrm{Fin}}_{query}$;3:**Similarity calculation by two-panel fingerprint-homogeneity model**4:**For** i = 1 to $N_{tr}$ do5:  Calculate the Euclidean distance and corresponding similarity of ${\mathrm{Fin}}_{query}$ and ${\mathrm{Fin}}_{train\;i}$ according to Equations (12) and (14)6:  Calculate the cosine distance and corresponding similarity of ${\mathrm{Fin}}_{query}$ and ${\mathrm{Fin}}_{train\;i}$ according to Equations (13) and (15)7:**End for**8:Sort $sim_{\mathrm{Euc}}$ in descending order, return the ${\mathrm{index}}_{\mathrm{Euc}}$;9:Sort $sim_{\cos}$ in descending order, return the ${\mathrm{index}}_{\cos}$;10:**SPSO Initialization**11:Set constants $N_{ps}$ = 100, *t* = 0, $T_{max}$ = 10,000, $c_{1}$ = $c_{2}$ = 1.5, $\omega_{init}$ = 0.4, $\omega_{end}$ = 0.9;12:Set boundary of the particle positions and velocities13:**For** each particle14:  Randomly initialize the particle positions $x_{i}^{0}$;15:  Randomly initialize the particle velocities $v_{i}^{0}$;16:  Evaluate the *i*th particle according to Equations (16)–(18) and set ${\mathrm{pbest}}_{i}^{0}=x_{i}^{0}$17:**End for**18:${\mathrm{gbest}}^{0}=\arg\;\min\left[f\left({\mathrm{pbest}}_{i}^{0}\right)\right]$19:**Particle swarm update process**20:**While**$t<=T_{max}$21:  $t=t+1,\omega^{t}=\omega_{init}+\left(\omega_{end}-\omega_{init}\right)\left(T_{\max}-t\right)/T_{\max}$22:  **For** each particle23:   Update $v_{i}^{t}$ and $x_{i}^{t}$ according to Equations (2) and (3)24:   Evaluate the *i*th particle according to Equations (16)–(18)25:   **If** $f(x_{i}^{t})$ < $f({\mathrm{pbest}}_{i}^{t-1})$26:    ${\mathrm{pbest}}_{i}^{t}=x_{i}^{t}$27:   **Else**28:    ${\mathrm{pbest}}_{i}^{t}={\mathrm{pbest}}_{i}^{t-1}$29:   **End if**30:  **End for**31:  **If** $\min[f\left({\mathrm{pbest}}_{i}^{t}\right)]$ < $f({\mathrm{gbest}}^{t-1})$32:   ${\mathrm{gbest}}^{t}=\arg\;\min\left[f\left({\mathrm{pbest}}_{i}^{t}\right)\right]$33:  **Else**34:   ${\mathrm{gbest}}^{t}={\mathrm{gbest}}^{t-1}$35:  **End if**36:**End while**37:**Return**gbest38:**Location estimation**39:$\left(x_{p},y_{p}\right)$ = (x,y) of gbest40:**Error evaluation**

## 4. Experiments and Analysis

### 4.1. Experimental Setup

**Figure 2:**
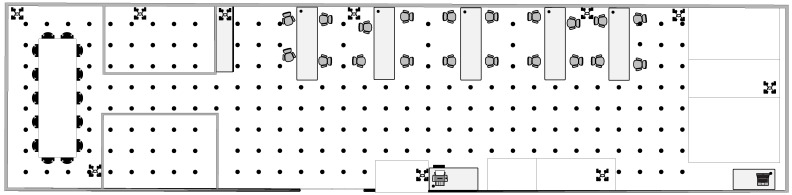
Layout of the experiment area (the dots represent the locations of all reference points).

**Figure 3:**
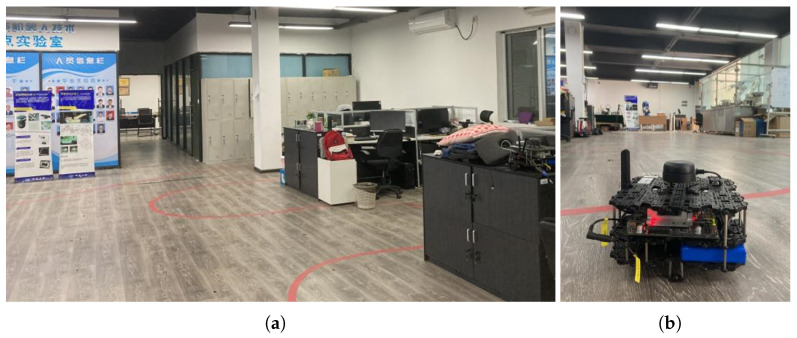
(**a**) The indoor environment. (**b**) The data-collection device.

The experiment was conducted in a 324 m${}^{2}$ one-floor building, with lengh of 27 m and width of 12 m. There are two offices, a conference room, an open office area containing five desks and several chairs, and an exhibition area containing six large robots, with relatively high but unintentional and random personnel flow. The spatial layout and indoor localization environment are shown in Figure 2 and Figure 3a, which include 10 APs deployed on the perimeter at a height of 1.2 m above the floor level. Notwithstanding, these APs are also shown, although their coordinates are not necessarily a priority condition. In addition, the data was collected by a mobile robot (product name: TurtleBot 3) with a RTL8188CUS Wi-Fi Module, as Figure 3b shows. To reflect the actual scenario, the data collection was performed in the presence of obstacles.

The localization area was divided into multiple grids of width 1.0 m. In the offline phase, 187 points were set as the RPs. At each RP, the RSS from each AP was uniquely identified by MAC address and measured 30 times (at 1 min intervals). The IEEE 802.11n with 2.4 GHz band, 40 MHz channel bandwidth and MCS 0, were used during this time. Thus, 56,100 units of RSS data were processed. The mean of each 30 measurements from the 10 APs were taken as the fingerprint of the RP. Further, the approximately uniformly distributed 52 groups of RPs with over 2.0 m spacing, and the corresponding fingerprints, constituted the training dataset, a sparse set; the remaining 135 groups constituted the test dataset and were used to test the performance of the localization system during the localization process.

### 4.2. Performance Metric

To evaluate the localization performance, several evaluation indices in machine learning were applied. The mean squared error (MSE), mean absolute error (MAE), root MSE (RMSE), and standard deviation (STD) were adopted as the main performance metrics. Furthermore, the mean of the Euclidean distance between the estimated location and the actual location was considered as a measure of accuracy. These metrics are defined as follows.

(19) $${\mathrm{Error}}_{MSE}=\frac{1}{N_{te}}\sum_{i=1}^{N_{te}}[{\left(x_{p\;i}-x_{test\;i}\right)}^{2}+{\left(y_{p\;i}-y_{test\;i}\right)}^{2}]$$

(20) $${\mathrm{Error}}_{MAE}=\frac{1}{N_{te}}\sum_{i=1}^{N_{te}}\left(|x_{p\;i}-x_{test\;i}|+|y_{p\;i}-y_{test\;i}|\right)$$

(21) $${\mathrm{Error}}_{RMSE}=\sqrt{\frac{1}{N_{te}}\sum_{i=1}^{N_{te}}\left[{\left(x_{p\;i}-x_{test\;i}\right)}^{2}+{\left(y_{p\;i}-y_{test\;i}\right)}^{2}\right]}$$

(22) $$\begin{array}{cc} {\mathrm{Error}}_{STD} & =\sqrt{\frac{1}{N_{te}}\sum_{i=1}^{N_{te}}{\left(err_{i}-\bar{err}\right)}^{2}},\\ err_{i} & =\sqrt{{\left(x_{p\;i}-x_{test\;i}\right)}^{2}+{\left(y_{p\;i}-y_{test\;i}\right)}^{2}},\\ \bar{err} & =\mathrm{Accuracy}=\frac{1}{N_{te}}\sum_{i=1}^{N_{te}}err_{i} \end{array}$$

where $\left(x_{p},y_{p}\right)$ is the estimated location obtained by the localization algorithm and $\left(x_{test},y_{test}\right)$ is the actual location of the RSS collection device.

### 4.3. Results and Discussion

#### 4.3.1. Performance Comparison of Different Methods

To evaluate the localization performance of the proposed system, it was compared with four other classical machine-learning (ML) algorithms: (1) the K-nearest-neighbor (KNN) method [44]; (2) the support-vector-machine (SVM) method [45]; (3) the linear-regression (LR) method [46]; and (4) the random-forest (RF) method [47]. All the ML-based results were calculated on a computer with 16.0 GB of RAM, Intel(R) Core(TM) i7-10700 CPU and the program environment of Python 3.7.8.

**Figure 4:**
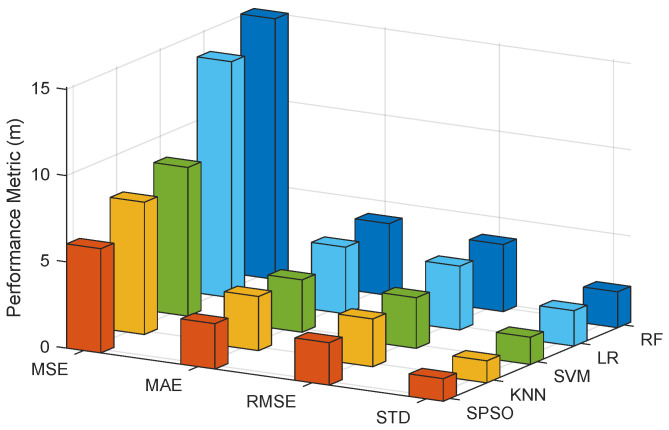
The MSE, MAE, RMSE and STD with different localization models.

A quantitative analysis of the localization errors was performed, as shown in Table 2. It shows that the four performance metrics of the proposed localization system were all minimum except for the STD, with MSE 6.0433 m, MAE 2.6288 m, and RMSE 2.4583 m, which is also shown in Figure 4. However, the difference in STD from the minimum was less than 0.04. Meanwhile, it is evident that it exhibited the best performance on the basis of 25/50/75% error, implying that the percentage accounted for all localization errors. In particular, the 50% error was less than 2.00 m and the 75% error was within 3.00 m. In terms of the improvement rate of RMSE, improvements by 11.25 16.28 33.56 and 36.76% compared with KNN, SVM, LR and RF, respectively, were observed. In general, the proposed method exhibited the best performance.

**Figure 5:**
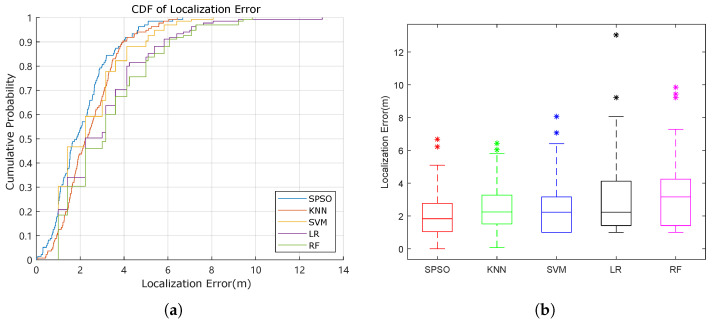
Distribution of the localization error. (**a**) CDF curve of the different models. (**b**) Box plot of the localization errors.

The CDF curve and the box plot can represent the localization performance and the distribution of the localization errors, in a visualized manner, as in Figure 5a,b, respectively. It is clear that the performance of SPSO is better than that of the other four. For LR and RF, their performances were not that different and were relatively mediocre. However, the performances of KNN and SVM were moderate. In the box plot, it is evident that, regardless of the median, maximum, minimum, upper quartile, or lower quartile, the localization error of the proposed method is the lowest, and no extreme outliers exist (outliers are shown as * in Figure 5b). Moreover, it still performs well in the presence of mild outliers.

For the accuracy, the method proposed in this study achieved 2.0817 m, which is the best out of the five localization models. Compared with the four conventional methods, the accuracy improved by 15.32%, 15.91%, 32.38%, and 36.64%, respectively. Considering the stochasticity of the SPSO algorithm, the proposed method was run 50 times with 100 particles and 10,000 iterations each time, and the above results are their average performance. The standard deviation of the accuracy over 50 runs was 0.0431 m. On the other hand, the SPSO algorithm inevitably increases the complexity of the system, conforming to the no-free-lunch theorem. A time-consumption comparison experiment was performed. For completing a single localization, all four conventional methods took less than 0.1 s, while SPSO took less than 0.05 s, which is also a real-time and acceptable result.

#### 4.3.2. Model Analysis

It should be noted that there are three factors that determine the performance of the model in the proposed method. In this section, comparison of one panel and two-panel, impact of different distance metrics, and impact of different weight assignations are discussed and analyzed. For the one panel, the fitness function Equation (18) was replaced by Equation (23). For the distance metrics, Euclidean metric (Euc, Equation (14)) and Mahalanobis distance (Mahal, Equation (24); the covariance matrix Σ was calculated by the training fingerprints) are common for Wi-Fi fingerprint similarity characterization. The correlation metric (Cor, Equation (25)) and cosine distance (Cos, Equation (15)) were adopted in [19]. For the weight assignments, reciprocal distance (weight 1 in Table 3) and Softmax function (weight 2 in Table 3) are commonly used. Considering that, in the log-distance path-loss model, the relationship between the RSS and the distance is related to the logarithmic function of base 10 [35], $\omega_{3}$ in Equation (26) (weight 3 in Table 3) was used in this study. The details are shown in Table 3.

(23) $$\begin{array}{c} f\left(x,y,K\right)=\sqrt{{\left(x-\sum_{k=1}^{K}\frac{\omega_{\;k}}{\sum_{k=1}^{K}\omega_{\;k}}x_{train}^{sim_{}k}\right)}^{2}+{\left(y-\sum_{k=1}^{K}\frac{\omega_{\;k}}{\sum_{k=1}^{K}\omega_{\;k}}y_{train}^{sim_{}k}\right)}^{2}} \end{array}$$

(24) $$dis_{\mathrm{Mahal}}\left({Fin}_{test},{Fin}_{train}\right)=\sqrt{\left({Fin}_{test}-{Fin}_{train}\right)\Sigma^{-1}{\left({Fin}_{test}-{Fin}_{train}\right)}^{T}}$$

(25) $$dis_{\mathrm{Cor}}\left({Fin}_{test},{Fin}_{train}\right)=1-\frac{1}{M-1}\sum_{j=1}^{M}\frac{\left(RSS_{test}^{AP\;j}-\bar{Fin_{test}})\cdot (RSS_{train}^{AP\;j}-\bar{Fin_{train}}\right)}{\sigma_{Fin_{test}}\cdot \sigma_{Fin_{train}}}$$

in which $\bar{\;•\;}$ and σ are, respectively, the mean and standard deviation of the fingerprint.

(26) $$\omega_{1k}=\frac{\frac{1}{d_{k}+\varepsilon }}{\sum_{k=1}^{K}\frac{1}{d_{k}+\varepsilon }},\;\;\omega_{2k}=\frac{e^{-d_{k}}}{\sum_{k=1}^{K}e^{-d_{k}}},\;\;\omega_{3k}=\frac{10^{-d_{k}}}{\sum_{k=1}^{K}10^{-d_{k}}}$$

in which $d_{k}$ means the distance between ${Fin}_{test}$ and $Fin_{train}^{sim\;k}$, and ε is a very small value to avoid the problem of division by zero.

*A. Comparison of one panel and two-panel.* To further analyze the characteristics of the model, a comparative experiment on one panel and two-panel was carried out. In this study, the two-panel fingerprint-homogeneity model was used to construct the fitness function of the SPSO algorithm. Actually, Equation (18) presents the fact that its geometric meaning is to find the situation where the estimated locations of the two panels are the closest. The situation can be determined by the parameter K, i.e., a specific value of K can uniquely determine the estimated locations of the two panels. For particles, their optimal position is on the line segment where the estimated location of the two panels are the endpoints. Obviously, Equation (18) has multiple solutions. However, if only one of the two panels were used, the particles would always find the optimal position that minimizes the fitness function (to 0), no matter what the value of K is. In the same case of multiple solutions, the solutions of one panel will be more dispersed, meaning that the model is not robust enough. As shown in Table 3, No. 1–4 are the results of one panel, and No. 5–10 are those of two-panel with different distance metrics and weight assignments. Although their results were similar, since *K* was limited to [2, 8] for better results, the performance of two-panel is better than at least one panel, in general. This means that the results of two panels can constrain each other, especially in combinations involving Mahalanobis distance.

*B. Impact of different distance metrics.* Wi-Fi fingerprint-based indoor localization is inseparable from the comparison of similar fingerprints. It is clear that different distance-characterization methods will lead to different localization results with the two-panel fingerprint-homogeneity model. A comparative experiment was conducted to analyze their impact on localization performance. The reliable covariance matrix cannot be obtained from the sparse training set; we can see in Table 3 that the accuracy of the combination involving Mahalanobis distance performs poorly. The two-panel method using Euclidean metric and cosine distance achieved the best performance. This is why we used them for Wi-Fi fingerprint-similarity characterization in this study.

*C. Impact of different weight assignments.* In fact, the neighboring fingerprints can generally be found correctly through different distance metrics. However, achieving high-accuracy localization with proper weight assignment is a challenging problem, because the transformation relationship from the fingerprint domain to the physical coordinate domain is uncertain. The effects of three weight assignments (Equation (26)) were compared experimentally in this study. Obviously, weight 1 is often applied, but it is not always the best. Weight 2 and weight 3 have little difference in actual performance. For the proposed method, weight 3 performs best with the two-panel approach using Euclidean metric and cosine distance. It verified the viability of weight 3 in Wi-Fi fingerprint-based indoor localization.

## 5. Conclusions

Although indoor localization based on Wi-Fi is promising, achieving improved accuracy remains a difficult problem. In this study, an application method of a particle swarm optimization algorithm in Wi-Fi fingerprint location was proposed, which adopted a two-panel fingerprint-homogeneity model to express the similarity among different fingerprints with greater robustness. The experimental results showed that the average accuracy of the proposed localization system was 2.0817 m. Further, the proposed particle swarm optimization algorithm outperforms other conventional algorithms, verifying its effectiveness and feasibility for improving the accuracy of indoor localization.

Future work will focus on extending radio fingerprint maps and mitigating the effects of Wi-Fi signal volatility. In addition, fusion localization with other methods will be considered to improve the performance of the localization system by combining the advantages offered by the different sensors.

## Figures and Tables

**Table 1:** The descriptions of each notation.

Notations	Descriptions
AP	Set of Access Points
*M*	Number of Access Points
${\mathrm{RP}}_{train},{RP}_{train\;i}$	Set of Reference Points for Training and its *i*th Point
${\mathrm{RP}}_{test},{RP}_{test\;i}$	Set of Reference Points for Test and its *i*th Point
${\mathrm{Fin}}_{train},{Fin}_{train\;i}$	Training Fingerprints and the *i*th Training Fingerprint
${\mathrm{Fin}}_{test},{Fin}_{test\;i}$	Test Fingerprints and the *i*th Test Fingerprint
$RSS_{train\;i}^{AP\;j}$	Received Signal Strength of *i*th RP from *j*th AP
$N_{tr}$	Number of Training Fingerprints
$N_{te}$	Number of Test Fingerprints/Query Fingerprints
${\mathrm{Fin}}_{query}$	The Query Fingerprint in the Online Phase
${dis}_{\mathrm{Euc}},{dis}_{\cos}$	Euclidean and Cosine Distance of Two Vectors
${sim}_{\mathrm{Euc}},{sim}_{\cos}$	The Simlarity Characterization of Two Vectors according to Euclidean and Cosine Distance
*K*	Number of the Most Similar Training Fingerprints to Test Fingerprint
${\mathrm{Fin}}_{train}^{sim\;k}$	The *k*th Most Similar Training Fingerprint to Query Fingerprint
$\omega_{\mathrm{Euc}\;k},\omega_{\cos\;k}$	The Location Coefficients of Location Estimation according to Euclidean and Cosine Distance
*D*	Dimension of the Particles
$N_{ps}$	Size of Particle Swarm/Number of Particles
$c_{1},c_{2}$	Acceleration Factors in SPSO
$\omega^{t}$	Inertia weight for the *t*th Iteration in SPSO
$v_{i}^{t},v_{id}^{t}$	Velocity vector and its *d*th Dimension Velocity of the *i*th particle in the *t*th iteration
$x_{i}^{t},x_{id}^{t}$	Position vector and its *d*th Dimension Position of the *i*th particle in the *t*th iteration
${\mathrm{pbest}}_{i}^{t},pbest_{id}^{t}$	Historical Optimal Solution of Each Particle and its *d*th Dimension Value in the *t*th iteration
${\mathrm{gbest}}^{t},gbest_{d}^{t}$	Historical Global Optimal Solution of Particle Swarm and its *d*th Dimension Value in the *t*th iteration
$T_{\max}$	Maximum Iterations

**Table 2:** The performance metrics of different localization models.

Performance Metrics	SPSO	KNN	SVM	LR	RF
MSE (m)	6.0433	7.6718	8.6224	13.6885	15.1111
MAE (m)	2.6288	3.1389	3.0370	3.8667	4.1630
RMSE (m)	2.4583	2.7698	2.9364	3.6998	3.8873
STD (m)	1.3076	1.2758	1.5789	2.0518	2.0777
Accuracy (m)	2.0817	2.4585	2.4757	3.0788	3.2855
25% Error (m)	1.0122	1.5104	1.0000	1.4142	1.4142
50% Error (m)	1.8329	2.2451	2.2361	2.2361	3.1623
75% Error (m)	2.7831	3.2639	3.1623	4.1231	4.2426
Improvement in RMSE	/	11.25%	16.28%	33.56%	36.76%
Improvement in Accuracy	/	15.32%	15.91%	32.38%	36.64%
Time Consumption (s)	<0.05	<0.01	<0.001	<0.001	<0.01

**Table 3:** The performance comparison ^1^ of different distance metrics and weight assignments.

No.	Distance Metrics	Weight	Accuracy (m)	RMSE (m)	STD (m)
1	Euclidean Metric	1	2.3600	2.6768	1.2631
		2	2.2261	2.6311	1.4026
		3	2.3555	2.7770	1.4708
2	Mahalanobis Distance	1	4.0090	4.7974	2.6350
		2	3.8229	4.5652	2.4952
		3	3.6852	4.5175	2.6128
3	Correlation Metric	1	2.3250	2.6555	1.2831
		2	2.4336	2.7733	1.3299
		3	2.4509	2.8180	1.3909
4	Cosine Distance	1	2.2089	2.5444	1.2629
		2	2.3378	2.6648	1.2789
		3	2.3857	2.7082	1.2818
5	Euc and Mahal	1	2.6692	3.1911	1.7490
		2	2.4599	2.9309	1.5934
		3	2.4903	3.0561	1.7714
6	Euc and Cor	1	2.2177	2.5252	1.2077
		2	2.1432	2.5172	1.3203
		3	2.1707	2.5491	1.3364
7	Euc and Cos	1	2.2584	2.5892	1.2664
		2	2.1516	2.5497	1.3681
		3	2.1128	2.4766	1.2922
8	Mahal and Cor	1	2.5656	3.0026	1.5599
		2	2.6912	3.1527	1.6422
		3	2.6900	3.2157	1.7621
9	Mahal and Cos	1	2.5490	2.9419	1.4689
		2	2.7242	3.1993	1.6775
		3	2.6901	3.2255	1.7797
10	Cor and Cos [19]	1	2.2559	2.5988	1.2902
		2	2.2831	2.6071	1.2587
		3	2.3244	2.6445	1.2612

^1^ All results in this table are medians over 20 runs.

## Data Availability

Not applicable.

## Abbreviations

The following abbreviations are used in this manuscript:

ANN	Artificial neural network
AOA	Angle of arrival
AP	Access point
GPS	Global positioning system
IMU	Inertial measurement units
IoT	Internet-of-Things
KNN	K-nearest neighbor
LR	Linear regression
MAE	Mean absolute error
MSE	Mean square error
NLOS	Non-line of sight
NN	Neural network
PSO	Particle swarm optimization
RF	Random forests
RFID	Radio frequency identification
RMSE	Root mean square error
RP	Reference point
RSS	Received signal strength
SPSO	Standard particle swarm optimization
STD	Standard deviation
SVM	Support vector machine
TDOA	Time difference of arrival
TOA	Time of arrival
UWB	Ultra wide band
WKNN	Weighted k-nearest neighbor
